# Supplementary material for: Novel but stable endosymbionts have contrasting effects on aphid dispersal and plant feeding damage in the cereal pest Diuraphis noxia
Source: eLife. 2026 Oct 21;15:RP111255. doi: 10.7554/eLife.111255 (PMC13686616; doi:10.7554/eLife.111255)
Supplement: Supplementary file 2 [file elife-111255-supp1.docx]

**Supplementary File 1. Criteria for aphid infestation and plant damage scores**

| **Score** | **Aphid infestation criteria** | **Plant damage criteria** |
| --- | --- | --- |
| 0 | No or all dead aphids * | As healthy as plants with no aphids |
| 1 | <20% total leaf area infested with alive aphids | Plant erect, with >80% leaves alive |
| 2 | 20-49% total leaf area infested with alive aphids | Plant erect, with 50-80% leaves alive |
| 3 | 50-79% total leaf area infested with alive aphids | Plant beginning to fall over, with <50% leaves alive |
| 4 | 80-99% total leaf area infested with alive aphids | Plant fallen over, with <20% leaves alive |
| 5 | 100% of total leaf area infested with alive aphids | Plant dead |

Note: dead plants with no aphids or only containing dead aphids were scored as 0.
