# Supplementary material for: Novel but stable endosymbionts have contrasting effects on aphid dispersal and plant feeding damage in the cereal pest Diuraphis noxia
Source: eLife. 2026 Oct 21;15:RP111255. doi: 10.7554/eLife.111255 (PMC13686616; doi:10.7554/eLife.111255)
Supplement: Peer review history

# Editor report: eLife Assessment

**DOI:** 10.7554/eLife.111255.3.sa0

**Authors:** Sergio Rasmann

**Affiliations:** University of Neuchâtel Switzerland

**Keywords:** Important, Solid

This **important** study investigates how experimentally introducing two facultative bacterial endosymbionts into the Russian wheat aphid, *Diuraphis noxia*, affects aphid performance, dispersal, and damage to cereal host plants and the authors provide **solid** evidence for contrasting symbiont-associated phenotypes under controlled conditions: *Rickettsiella* increases plant damage while reducing wing production and dispersal, whereas *Regiella* reduces feeding damage and, at some time points, aphid population growth. The stable establishment of these novel symbiont-host associations and complementary experiments spanning individual aphids, whole plants, populations, and mesocosms are notable strengths and the revised manuscript better clarifies the experimental approaches and the context dependence of symbiont effects; however, the underlying mechanisms remain unresolved, horizontal transmission is inferred rather than directly demonstrated, and limited replication and temporal variability constrain some population-level conclusions. Potential applications to pest management therefore require further validation under field conditions. The study will interest researchers working on insect symbiosis, plant-insect interactions, and biologically based pest management.

# Referee report: Reviewer #1 (Public review)

**DOI:** 10.7554/eLife.111255.3.sa1

[Editors' note: this version has been assessed by the Reviewing Editor without further input from the original reviewers. The authors have addressed the comments raised in the previous round of review.]

Summary:

In this study, the authors examine what happens when two facultative endosymbionts, *Rickettsiella viridis* and *Regiella insecticola*, are introduced into a novel aphid host, the Russian wheat aphid (*Diuraphis noxia*). They ask whether these introduced symbionts affect aphid performance, plant damage, alate production, dispersal, plant defense responses, and symbiont dynamics. The main result is that the two symbionts have contrasting effects: *Rickettsiella* tends to increase plant damage and reduce dispersal-related traits, whereas Regiella tends to reduce plant damage and aphid population growth, with less evidence for an effect on dispersal.

Strengths:

The manuscript presents successful establishment of stable transinfected populations of an agriculturally important aphid species, which is a substantial technical achievement in itself. I also appreciated that the authors examined the system across several experimental contexts, including different host plants, mixed cages at two temperatures, whole-plant assays, and a mesocosm dispersal experiment, rather than relying on a single laboratory setup. Taken together, these experiments provide a useful and reasonably convincing demonstration that novel symbiont associations can generate contrasting phenotypes in this system.

# Referee report: Reviewer #2 (Public review)

**DOI:** 10.7554/eLife.111255.3.sa2

Summary:

The authors generated two novel aphid-symbiont associations and examined the impact of these new symbiotic associations on plant-insect-symbiont interactions. The authors notably provide detailed phenotypic assessments of the insect hosts and host plants. They show that one introduced symbiont, *Rickettsiella*, increases aphid-induced damage to host plants, while the other, Regiella, ameliorates aphid damage. The authors suggest that such novel insect-symbiont pairings may be used as tools to mitigate crop damage in the future.

Strengths:

Although a few experiments seem to have limited sample sizes and limited statistical power, these are often complemented with highly replicated smaller-scale experiments. The combination of larger mesocosm and population-level experiments along with assessments of individual insects generally provides a comprehensive depiction of the effects of these symbionts on their hosts. The opposing impacts of Regiella and *Rickettsiella* infection on the aphid host plant are of broad interest. It is also surprising that the host plants did not exhibit strong differences in canonical defensive signalling, despite these differences.

Weaknesses:

It is a little surprising that mesocosm-dispersal experiments were not also conducted using Regiella-infected lines. At several points throughout the manuscript, the idea of using symbiont transfections to reduce plant harm is raised. I can understand that these experiments are likely time-, space-, and resource-intensive, but that seems like these would have been relevant experiments, especially in the context of controlling damage to plants.

# Referee report: Reviewer #3 (Public review)

**DOI:** 10.7554/eLife.111255.3.sa3

Summary:

The authors were investigating the impact of introducing novel facultative bacterial endosymbionts into the pest aphid, *Diuraphis noxia*, to explore the possibility of using facultative symbionts as a crop protection tool. They successfully established the vertical transmission of both endosymbionts and performed a series of aphid performance and dispersal experiments together with measurement of aphid feeding on host plant health, growth, and metabolism. While most of the experiments revealed no effect of the endosymbionts, some significant treatment effects were found, showing that *Rickettsiella* reduced aphid dispersal, and Regiella reduced aphid population growth and feeding damage.

Strengths:

The team worked with two novel facultative symbionts (*Rickettsiella viridis* and *Regiella insecticola*) that they were able to successfully establish in D. noxia. The data were collected and analyzed using solid, well-described methodology.

Weaknesses:

While interpretation of the data is reasonable, the few experiments which revealed significant treatment effects rest on relatively small sample sizes.

Measuring symbiont density is difficult. The authors use quantitative PCR to measure the "density" of endosymbionts relative to a host gene. This is a standard approach in the field; however, recent work has shown that endosymbionts like the aphid primary endosymbiont, Buchnera, are variably polyploid [1]; further the aphid cells that house the symbionts are also highly polyploid and variable in their ploidy [2]. It is important to understand that what is being measured when using qPCR is DNA copy number and not quantification of the number of symbiont cells. Alternative approaches to measuring symbiont density include flow cytometry [3], and SymbiQuant [4], a machine vision tool that can quantitatively characterize symbiont populations from DAPI-stained confocal images. These alternate approaches also have their limitations. Currently, there is no perfect approach to measuring symbiont density, which remains an important measure in experiments such as these. Put simply, it is important for a reader to be aware of the limitations of each approach and interpret data accordingly.

[1] Komaki, K., and H. Ishikawa. 2000. Genomic copy number of intracellular bacterial symbionts of aphids varies in response to developmental stage and morph of their host. Insect Biochemistry and Molecular Biology 30:253-258.

[2] Nozaki, T., and S. Shigenobu. 2022. Ploidy dynamics in aphid host cells harboring bacterial symbionts. Scientific Reports 12:9111.

[3] Simonet, P., G. Duport, K. Gaget, M. Weiss-Gayet, S. Colella, G. Febvay, H. Charles, J. Viñuelas, A. Heddi, and F. Calevro. 2016. Direct flow cytometry measurements reveal a fine-tuning of symbiotic cell dynamics according to the host developmental needs in aphid symbiosis. Scientific Reports 6:19967.

[4] James, E. B., X. Pan, O. Schwartz, and A. C. C. Wilson. 2022. SymbiQuant: A machine learning object detection tool for polyploid independent estimates of endosymbiont population size. Frontiers in Microbiology 13:816608.

Impact and Significance:

Food security and production, and pest control are major challenges facing the human population. This work contributes knowledge that will benefit the development of alternate pest control strategies in agriculture.

# Author comment: Author response

**DOI:** 10.7554/eLife.111255.3.sa4

**Authors:** Xinyue Gu, Alex Gill, Qiong Yang, Perran Ross, Lucy Hayward, Monica Stelmach, Paul A Umina, Sheik Nadeem Elahee Doomun, Mel Berran, Lauren Coakley, Sonia Sharma, Ary Hoffman

**Affiliations:** The University of Melbourne Parkville Australia; The University of Melbourne Parkville Australia; The University of Melbourne Parkville Australia; The University of Melbourne Parkville Australia; The University of Melbourne Parkville Australia; The University of Melbourne Parkville Australia; The University of Melbourne Parkville Australia; The University of Melbourne Parkville Australia; The University of Melbourne Parkville Australia; The University of Melbourne Parkville Australia; The University of Melbourne Parkville Australia; The University of Melbourne Parkville Australia

The following is the authors’ response to the original reviews.

**eLife Assessment:**

This important study investigates how experimentally introducing two facultative bacterial endosymbionts into the Russian wheat aphid, *Diuraphis noxia*, affects aphid performance, dispersal, and damage to cereal host plants. The authors provide solid evidence that the two symbionts can generate contrasting phenotypes: *Rickettsiella* increases plant damage while reducing wing formation and dispersal, whereas *Regiella* reduces aphid population growth and feeding damage. The successful establishment and stable transmission of these novel symbiont-host associations, combined with experiments spanning individual, whole-plant, population and mesocosm scales, are notable strengths of the work; however, the mechanisms underlying these effects remain unresolved, evidence for horizontal transmission is indirect, and some population-level conclusions rely on relatively small sample sizes or effects that are not consistently detected across time points, and therefore the potential application of these findings to pest management remains promising but speculative. The study will be of broad interest to researchers working on insect symbiosis, plant-insect interactions, and biologically based approaches to pest management.

We have made revisions to the manuscript to cover issues raised around sample numbers and mechanisms. We appreciate that we have not been able to finalize the exact mechanism underlying plant damage effects. We note that while comparisons of population performance were limited by the number of populations we could feasibly maintain; sample sizes were substantial in some of the other experiments. We also do substantiate effects through a combination of experimental approaches that start with controlled conditions and then encompass more complex environments.

**Public Reviews:**

**Reviewer #1 (Public review):**

Summary:

In this study, the authors examine what happens when two facultative endosymbionts, *Rickettsiella viridis* and *Regiella insecticola*, are introduced into a novel aphid host, the Russian wheat aphid (*Diuraphis noxia*). They ask whether these introduced symbionts affect aphid performance, plant damage, alate production, dispersal, plant defense responses, and symbiont dynamics. The main result is that the two symbionts have contrasting effects: *Rickettsiella* tends to increase plant damage and reduce dispersal-related traits, whereas *Regiella* tends to reduce plant damage and aphid population growth, with less evidence for an effect on dispersal.

Strengths:

The manuscript presents successful establishment of stable transinfected populations of an agriculturally important aphid species, which is a substantial technical achievement in itself. I also appreciated that the authors examined the system across several experimental contexts, including different host plants, mixed cages at two temperatures, whole-plant assays, and a mesocosm dispersal experiment, rather than relying on a single laboratory setup. Taken together, these experiments provide a useful and reasonably convincing demonstration that novel symbiont associations can generate contrasting phenotypes in this system.

We thank the reviewer for recognizing the strengths of the work and for providing extensive comments.

Weaknesses:

There are some major aspects of this paper that I thought could be strengthened. My main concern is that the manuscript feels broader than it is conceptually focused. A wide range of outcomes is measured, which gives the study breadth, but it also makes the central question harder to identify. As written, the paper reads more strongly as a proof-of-principle demonstration of ecologically relevant phenotypes than as a tightly framed test of a specific biological idea.

The broad range of tests in our work was intentional. Rather than relying on a single experimental approach or scale, we designed the study using multiple complementary approaches to independently evaluate the effects of endosymbionts. Thus, our conclusions are supported across multiple experimental contexts rather than by a single experiment or scale. For example, the effects on plant damage were consistent across different host plants, including wheat and barley (Figures 1 & Figure 1—figure supplement 1), and across different experimental scales, ranging from individual plants maintained in small cages (Figure 1) to a dispersal experiment involving 24 plants (Figure 5). Similarly, aphid fitness was evaluated both at the individual level, using single aphids maintained on individual plants under favorable conditions (Figure 3—figure supplement 1 & Figure 4—figure supplement 1), and at the population level under crowding conditions (Figures 3 & 4).

Despite the breadth of measurements, the study is focused on establishing robust evidence for the contrasting effects of the two endosymbionts on aphid dispersal and plant feeding damage. To help address this concern, we have moved the summary table from the Supplementary Materials to the main text (now Table 1), which provides an overview of the experimental approaches and main findings and should help readers more clearly see how the different experiments are connected.

A second issue is that the biological basis of the reported phenotypes remains less developed than the phenotypic description itself. The authors make a genuine effort to address mechanism through JA, JA-Ile, SA, and metabolomic profiling, but these analyses only partially explain the main results. The negative result for the canonical defense markers is informative, yet it still leaves a substantial gap between the observed variation in plant damage and the processes responsible for it.

Our analyses of JA, JA-Ile, SA, and the metabolomic profiles provide some initial insights, but we appreciate that they do not fully explain the differences in plant damage observed between treatments. The primary aim of this study was to evaluate the phenotypic effects of the endosymbionts and their potential for agricultural application, rather than to provide a comprehensive mechanistic explanation. We have accordingly avoided overinterpreting the mechanistic results and now explicitly state that elucidating the underlying biological mechanisms will be an important direction for future research in Discussion section.

I also think some caution is needed in how the two symbionts are compared. The authors explain why some follow-up experiments were designed differently for *Rickettsiella* and *Regiella*, and that rationale is understandable. Still, because the downstream assays were not fully matched, the paper is strongest when each symbiont is interpreted on its own terms rather than as a strict comparison.

Our initial plant-damage experiment was designed as a first comparison to test whether different endosymbionts can have diverse and contrasting effects on their aphid host population and plant damage. We then investigated the individual phenotypes of each endosymbiont in greater detail, particularly in relation to their potential agricultural applications. Specifically, our results suggest that *Regiella* may reduce plant damage, whereas *Rickettsiella* may reduce dispersal. Based on these early findings, some further experiments were conducted with slightly different experimental setups. Nevertheless, many of the experiments conducted for the two endosymbionts were broadly comparable. We designed the additional experiment carried out only with *Rickettsiella* to test whether reduced alate production observed in our earlier experiments also translated into reduced dispersal at the population level. An equivalent experiment with *Regiella* was not undertaken because we failed to detect an effect of this endosymbiont on alate frequency in our preceding experiments. We have clarified this rationale in the Materials and methods section (“Aphid dispersal ability and plant feeding damage in mesocosms”).

Overall, I would suggest softening the Significance Statement so that it more clearly reflects what is directly shown here, namely that introduced symbionts can alter plant damage and dispersal-related phenotypes under controlled conditions, rather than implying that the study directly tests management utility in agricultural settings.

We have done this in the Significance Statement. We appreciate that the current experiments have been carried out under controlled conditions, rather than in agricultural settings. Pending permit approval, we are currently planning to extend this work to contained field settings to test whether effects on plant damage and dispersal ability are also observed under less controlled conditions.

**Reviewer #2 (Public review):**

Summary:

The authors generated two novel aphid-symbiont associations and examined the impact of these new symbiotic associations on plant-insect-symbiont interactions. The authors notably provide detailed phenotypic assessments of the insect hosts and host plants. They show that one introduced symbiont, *Rickettsiella*, increases aphid-induced damage to host plants, while the other, *Regiella*, ameliorates aphid damage. The authors suggest that such novel insect-symbiont pairings may be used as tools to mitigate crop damage in the future.

Strengths:

Although a few experiments seem to have limited sample sizes and limited statistical power, these are often complemented with highly replicated smaller-scale experiments. The combination of larger mesocosm and population-level experiments along with assessments of individual insects generally provides a comprehensive depiction of the effects of these symbionts on their hosts. The opposing impacts of *Regiella* and *Rickettsiella* infection on the aphid host plant are of broad interest. It is also surprising that the host plants did not exhibit strong differences in canonical defensive signalling, despite these differences.

Weaknesses:

One thing that I struggled a little with was the rapid spread of *Regiella* in the shared plant experiments. Possibly this could be attributed to an increased reproductive output (due to faster developmental time, and/or an increase in fecundity) or efficient horizontal transmission. However, the other experiments performed indicate a slight negative impact (Figure 4a) or no influence (Figure 4C, 4D, Figure 4—figure supplement 1) of *Regiella* infection on host fitness. Given these other results, it seems that *Regiella* must spread fairly efficiently between hosts, which comes as a surprise, and there are very few examples of horizontal transmission of *Regiella* like this in the literature. The manuscript would benefit from a clear and direct demonstration of horizontal transmission, rather than it being inferred indirectly. The similar spread observed in the *Rickettsiella* mixed cages is less surprising, because there are several examples where this has been demonstrated.

We agree that the rapid spread of *Regiella* in the shared-plant experiments cannot be readily explained by host fitness alone, though we have noted fitness benefits of *Regiella* in a different transinfection in oat aphids (Yu et al., 2025). In a previous study with transinfected green peach aphids and despite a substantial fitness cost, we found that *Rickettsiella* can spread relatively rapidly in a population and show high stability (Gu et al., 2023) and perhaps transmission of *Regiella* follows a similar pathway. However, whereas *Rickettsiella* may spread through plant tissues, *Regiella* showed relatively low levels of horizontal transmission through this pathway.

We certainly agree that more work is required to establish the mechanism and dynamics of horizontal transmission in this system. Rather than focusing on mechanism, our objective here was to examine endosymbiont spread where intact plants were available and where there was a mixed aphid population. Note that we also conducted an additional experiment in which *Regiella*-infected aphids were present at a frequency of only 10% of the initial population, and in this situation *Regiella* nevertheless still increased in frequency including to a low Cp value in most (8/10) replicates, highlighting its persistence and potential to increase in populations.

References:

Gu et al., A rapidly spreading deleterious aphid endosymbiont that uses horizontal as well as vertical transmission. Proc Natl Acad Sci USA 120, e2217278120 (2023).

Yu et al., A persistent bacterial *Regiella* transinfection in the bird cherry-oat aphid *Rhopalosiphum padi* increasing host fitness and decreasing plant virus transmission. Pest Manag Sci 81, 2791-2799 (2025).

It is also a little surprising that mesocosm-dispersal experiments were not also conducted using *Regiella*-infected lines. At several points throughout the manuscript, the idea of using symbiont transfections to reduce plant harm is raised. I can understand that these experiments are likely time-, space-, and resource-intensive, but that seems like these would have been relevant experiments, especially in the context of controlling damage to plants.

We conducted the final mesocosm-dispersal experiment specifically with *Rickettsiella* because our earlier individual-plant experiments had already shown a clear reduction in alate production in *Rickettsiella*-infected aphids, together with effects on plant damage (Figure 1—figure supplement 1I) and population growth (Figure 3C). In contrast, we did not detect a significant difference in alate frequency between *Regiella*-infected and wild type aphid strains in the similar set up experiments (Figure 1—figure supplement 1I and Figure 4C). We therefore designed the additional experiment to test whether the reduced alate production observed with *Rickettsiella* also translated into reduced dispersal at the population level. We did not conduct the same experiment with *Regiella* because there was no difference in alate frequency in our earlier experiments. We have also added this explanation in Materials and methods section (“Aphid dispersal ability and plant feeding damage in mesocosms”). We do appreciate however that future experiments on dispersal of *Regiella* will be worthwhile resources permitting.

**Reviewer #3 (Public review):**

Summary:

The authors were investigating the impact of introducing novel facultative bacterial endosymbionts into the pest aphid, *Diuraphis noxia*, to explore the possibility of using facultative symbionts as a crop protection tool. They successfully established the vertical transmission of both endosymbionts and performed a series of aphid performance and dispersal experiments together with measurement of aphid feeding on host plant health, growth, and metabolism. While most of the experiments revealed no effect of the endosymbionts, some significant treatment effects were found, showing that *Rickettsiella* reduced aphid dispersal, and *Regiella* reduced aphid population growth and feeding damage.

Strengths:

The team worked with two novel facultative symbionts (*Rickettsiella viridis* and *Regiella insecticola*) that they were able to successfully establish in *D. noxia*. The data were collected and analyzed using solid, well-described methodology.

Weaknesses:

While interpretation of the data is reasonable, the few experiments which revealed significant treatment effects rest on relatively small sample sizes.

We acknowledge that more replication is always desirable, but we would also argue that significant effects were not marginal and replication was substantial in many cases (e. g. 9-10 replicate plants per damage treatment evaluation). We were also focused on using multiple experimental approaches and scales to independently and repeatedly evaluate the effects of endosymbionts on aphid fitness, wing development, plant damage, and aphid dispersal. Thus, conclusions are not based on a single experiment or experimental scale. For plant damage, for example, we observed consistent effects across different host plants, including wheat and barley (Figure 1 and Figure 1—figure supplement 1), as well as across different experimental scales, from individual plants maintained in small cages (Figure 1) to a dispersal experiment involving 24 plants (Figure 5). Similarly, aphid fitness was evaluated both at the individual level using single aphids maintained on individual plants under favorable conditions (60 replicates per treatment) (Figure 3—figure supplement 1 & Figure 4—figure supplement 1) and at the population level under crowding conditions (Figures 3 & 4). These complementary experimental designs allowed us to examine whether the observed phenotypes were consistent across different environmental and population contexts. We did face challenges in high levels of replication of independent aphid strains in population cage experiments but attempted to replicate as much as possible given the resources that were available.

Measuring symbiont density is difficult. The authors use quantitative PCR to measure the "density" of endosymbionts relative to a host gene. This is a standard approach in the field; however, recent work has shown that endosymbionts like the aphid primary endosymbiont, *Buchnera*, are variably polyploid [1]; further the aphid cells that house the symbionts are also highly polyploid and variable in their ploidy [2]. It is important to understand that what is being measured when using qPCR is DNA copy number and not quantification of the number of symbiont cells. Alternative approaches to measuring symbiont density include flow cytometry [3], and SymbiQuant [4], a machine vision tool that can quantitatively characterize symbiont populations from DAPI-stained confocal images. These alternate approaches also have their dlimitations. Currently, there is no perfect approach to measuring symbiont density, which remains an important measure in experiments such as these. Put simply, it is important for a reader to be aware of the limitations of each approach and interpret data accordingly.

(1) Komaki, K., and H. Ishikawa. 2000. Genomic copy number of intracellular bacterial symbionts of aphids varies in response to developmental stage and morph of their host. Insect Biochemistry and Molecular Biology 30:253-258.

(2) Nozaki, T., and S. Shigenobu. 2022. Ploidy dynamics in aphid host cells harboring bacterial symbionts. Scientific Reports 12:9111.

(3) Simonet, P., G. Duport, K. Gaget, M. Weiss-Gayet, S. Colella, G. Febvay, H. Charles, J. Viñuelas, A. Heddi, and F. Calevro. 2016. Direct flow cytometry measurements reveal a fine-tuning of symbiotic cell dynamics according to the host developmental needs in aphid symbiosis. Scientific Reports 6:19967.

(4) James, E. B., X. Pan, O. Schwartz, and A. C. C. Wilson. 2022. SymbiQuant: A machine learning object detection tool for polyploid independent estimates of endosymbiont population size. Frontiers in Microbiology 13:816608.

We agree that qPCR-based measurements of endosymbiont gene copy number have limitations even if they are the standard approach used in most studies. In the current set of experiments, qPCR provided a practical and efficient approach for assessing variation in endosymbiont abundance and infection status among samples and the only one feasible given the number of monitoring events and samples required. Nevertheless, we acknowledge the limitations of this approach (and have pointed this out ourselves in a recent COIS paper – Hoffmann et al 2026). We now mention this under further work and provide a couple of references (see Discussion).

Reference:

Hoffmann, A. A., Yang, Q. and P. A. Ross. Aphid endosymbionts revisited: molecular detection, diversity, and population dynamics. Curr Op Insect Sci (in press). (2026)

Impact and Significance:

Food security and production, and pest control are major challenges facing the human population. This work contributes knowledge that will benefit the development of alternate pest control strategies in agriculture.

**Recommendations for the authors:**

**Reviewer #1 (Recommendations for the authors):**

(1) Overall, I found the study interesting and worthwhile, particularly because it shows that novel symbiont associations can generate contrasting phenotypes in an important pest species. My main suggestion would be to sharpen the framing of the paper and to bring the mechanistic and applied discussion into slightly closer alignment with the current evidence base. With those points addressed, I think the manuscript would read as a clearer and more balanced contribution.

We have rephrased the Discussion around the mechanistic component of the work to sharpen this and link more directly to evidence. For instance:

“Despite this, our metabolomic analyses suggest that endosymbionts in *D. noxia* may influence some other aspects of wheat metabolism in a spatially structured way. Within aphid-feeding areas, wheat exposed to *Rickettsiella* aphids showed increased valine and decreased 3-phenyllactic acid. Changes in valine have been reported in plants responding to herbivory and other forms of stress (43-45), while 3‑phenyllactic acid has been associated with antimicrobial and defense-related activity (46). In non-feeding areas, wheat exposed to *Regiella* aphids showed reduced urea and increased pantothenic acid. These changes may reflect differences in nitrogen metabolism and allocation (47), and in metabolic processes involving pantothenic acid (48) respectively. However, the present metabolomic data do not establish the functional consequences or causal mechanisms underlying these changes. They indicate that aphids carrying different endosymbionts are associated with some distinct metabolic responses in wheat, including responses that differ between aphid-feeding and non-feeding areas. Our findings are consistent with previous work showing that phloem‑feeding insects can induce changes in plant metabolites in response to herbivory (49, 50) and provide a basis for future studies to determine how endosymbionts influence aphid-induced plant responses and contribute to contrasting plant phenotypes.”

We have not undertaken a complete reframing of the paper but further emphasized the focus on phenotypic contrasts in a few places including incorporating some changes to the comments below.

(2) Lines 130-135: Please clarify more explicitly whether the main aim of the paper is to test a specific biological hypothesis about endosymbiont-mediated aphid-plant interactions or to provide a broader proof-of-principle survey of symbiont-associated phenotypes. As it stands, the framing moves between multitrophic biology and pest-management relevance, which makes the central conceptual contribution harder to identify.

We have rephrased this sentence as “By integrating these factors, we investigate how endosymbiont infection influences aphid fitness and aphid–plant interactions, providing insights into the ecological consequences of novel microbial associations and their potential relevance to sustainable pest management.”

(3) Line 205: For the metabolomic analysis, please consider adding a formal multivariate test for strain effects, especially for the comparisons shown in Figure 2C and 2D, or otherwise interpret the PCA more cautiously as descriptive rather than inferential.

We have emphasized the descriptive component and rephrased this sentence as “However, within either area, there was no clear separation among aphid strains (Figures 2C & 2D), suggesting broadly similar metabolomic profiles among strains of the same aphid clone carrying different symbionts.”

(4) Please clarify how the top and bottom feeding leaves were handled analytically in the analyses, and explain the rationale for collapsing them into a single "feeding area" category. If possible, it would be helpful to show whether leaf position itself influenced the plant-response patterns.

We combined the upper and lower leaves together to provide a representative measure of the plant-level responses, rather than focusing on responses at a particular leaf position. This approach was consistent with the main objective of our study, which was to investigate whole plant responses to aphids hosting different endosymbionts, rather than differences in responses among different plant parts. In addition, combining the two portions provided sufficient plant material for the GC-MS analysis and helped ensure reliable metabolite measurements from the same material. Because the two leaf positions were combined prior to GC-MS analysis, we were unable to separately test the effect of leaf position on the metabolomic response in this dataset. We have clarified this point in the revised manuscript in Materials and methods section (“Plant defense responses”).

(5) Line 228: The use of 19{degree sign}C and 25{degree sign}C is not unusual in aphid work, but it would still help the reader if the manuscript stated more explicitly why these two temperatures were chosen in this study.

We selected 19°C and 25°C because they represent contrasting temperature conditions within the range suitable for Russian wheat aphid development, allowing us to assess whether temperature influences *Rickettsiella* transmission and population dynamics. In particular, our previous observations indicated differences in the rate of *Rickettsiella* spread between these temperature conditions (Gu et al., 2023).

Reference:

Gu et al., A rapidly spreading deleterious aphid endosymbiont that uses horizontal as well as vertical transmission. Proc Natl Acad Sci USA 120, e2217278120 (2023).

(6) Lines 390-392: It would help to discuss more explicitly how the relatively modest effects in the individual life-history assays relate to the clearer signals seen at the whole-plant and population level.

In our experiments, we assessed aphid fitness under different environmental conditions. In the individual fitness assays conducted on cups with a single plant (Figure 3—figure supplement 1 & Figure 4—figure supplement 1), aphids were maintained under relatively favourable conditions, with limited environmental stress and without substantial crowding. Under these conditions, we observed an increase in fitness associated with endosymbiont infection. In contrast, we also examined aphid performance at the population level (Figures 3 & 4), where populations were established from a small number of aphids and subsequently experienced increasing crowding and density-dependent stress. Under these conditions, the effects of endosymbiont infection differed from those observed in the individual assays, with *Rickettsiella*-infected aphids showing greater population growth and *Regiella*-infected aphids showing reduced population growth.

These results suggest that the effects of endosymbionts on aphid fitness are context-dependent and may become more pronounced as population density increases and density-dependent stress develops. Thus, relatively modest effects observed at the individual level where experiments are often undertaken may not translate to differences at the population level, and plant level effects may subsequently influence feeding pressure and plant damage. We have added this perspective in the Results and Discussion sections.

(7) Line 678 & 688: In both whole-plant experiments, please explain how 3 and 4 replicate plants were selected.

The replicate plants were randomly selected from the available plants for each treatment to minimize potential selection bias. We have clarified this procedure in the revised manuscript in Materials and methods section.

(8) Lines 686-690 / Figure 4: In the *Regiella* whole-plant experiment, the Methods state that 16 plants were established per treatment and that 4 plants per treatment were removed at each time point (days 4, 8, 12, and 16). However, in Figure 4B-D, day 12 appears to include 5 data points. Please clarify this apparent mismatch between the described sampling scheme and the data shown in the figure.

We thank the reviewer for pointing out this and we have corrected this mistake. We initially established 16 plants for the wild type and 17 plants for the *Regiella* treatment. Four plants per treatment were originally planned to be sampled at each time point (Days 4, 8, 12, and 16). However, because Day 12 was a key time point at which an obvious difference in plant damage was observed between the treatments, we selected one additional plant each treatment for measurement at Day 12, resulting in five data points for this treatment at that time point. The remaining plant was therefore measured at Day 16. We have clarified the sampling procedure in the revised Materials and methods section and figure legend.

(9) Figure 2A and Figure 4A: These schematics are helpful overall, but the brown supporting sticks stand out quite strongly and may make the panels a little harder to interpret at first glance. I wonder whether they could be simplified, made less prominent, or replaced with photographs of the actual setup if those are available.

We have revised Figures 2A and 4A to simplify the supporting structures and reduce their visual prominence.

**Reviewer #2 (Recommendations for the authors):**

Some of the statistics were not entirely clear to me, particularly the tests reported in the results which differ from what is described in the figure legends:

(1) Lines 306-308: "Total nymph numbers were higher on *Rickettsiella* aphids from Day 21 to Day 25" and indicates that this is based on GLM testing, but the figure does not indicate statistical significance, and the figure legend states independent sample t-tests were used. Similar comment for the following paragraph and corresponding figure.

The GLMs were used to test the overall patterns in nymph numbers across the relevant time periods, including Days 21–25, rather than testing each time point independently. We also conducted independent-sample t-tests to assess differences between treatments at individual time points. We have clarified this distinction in the Statistical section.

(2) Line 43: Does not seem like the appropriate reference (reference is on plant virus transmission, not salivary toxins).

We thank the reviewer for pointing this out. We have removed this reference and replaced it with reference 34 (Luna et al., 2018) that directly supports the statement regarding aphid salivary toxins.

Reference:

Luna et al., Bacteria associated with Russian wheat aphid (*Diuraphis noxia*) enhance aphid virulence to wheat. Phytobiomes J 2, 151-164 (2018).

**Reviewer #3 (Recommendations for the authors):**

Minor editorial comments:

(1) Figure 3—figure supplement 3 - the figure legend needs improvement as the current version does not help the reader understand the figure. Please also include a key.

We have changed the figure legend with reference to our aim, and also explained use of the Cp values. “Figure 3—figure supplement 3. *Rickettsiella* Cp values in (A) routine screening of laboratory *Rickettsiella* colonies and (B) the mixed cage experiment assessing endosymbiont frequency changes over time at 19 °C and 25 °C. The dark red area represents overlap between the 19°C and 25°C experiments. Cp values represent the quantification cycle values obtained from qPCR, with lower Cp values indicating a higher amount of *Rickettsiella* target DNA. This figure shows the typical range of Cp values observed in our laboratory *Rickettsiella* -infected aphid colonies. We used this range as a reference for identifying aphids that acquired *Rickettsiella* through horizontal transmission, as horizontally infected aphids generally showed much higher Cp values than vertically infected aphids.”

(2) Define Cp.

We have explained it as “Cp values represent the quantification cycle values obtained from qPCR, with lower Cp values indicating a higher amount of *Rickettsiella* target DNA.”

(3) Supplemental Information: Line 113 - T is missing from Table.

This has been added.

(4) Move Table S2 to the main paper - this table provides a useful summary of the work.

This has been moved.

Main Manuscript:

(1) Line 145: *Serratia* was not detected at G0 - was it detected later? It seems possible that titer could be very low to begin and increase in later generations; please clarify.

We have previously monitored the aphid populations for the presence of *Serratia* transinfected from the same donor resource across subsequent generations, and *Serratia* was not detected at any later generation. We also did not detect *Serratia* in the other aphid species we examined, including green peach aphids (Gu et al., 2023 & 2025) and oat aphids (Yang et al., 2026). Therefore, we believe that the absence of *Serratia* at G0 was not due to a very low initial titer followed by an increase in later generations but instead that *Serratia* had been lost from the aphid population.

References:

Gu et al., A rapidly spreading deleterious aphid endosymbiont that uses horizontal as well as vertical transmission. Proc Natl Acad Sci USA **120**, e2217278120 (2023).

Gu et al., Transinfections of the endosymbiont *Rickettsiella viridis* in different *Myzus persicae* (Hemiptera: Aphididae) clones show consistent deleterious effects and stable transmission. J Econ Entomol **118**, 1544-1552 (2025).

Yang et al., *A Rickettsiella* transinfection in *Rhopalosiphum padi* reduces fitness and alate production but not plant virus transmission. Pest Man Sci **82**, 3894-3906 (2026).

(2) Line 206: "different aphid strain" - I learned from the manuscript that a single clone of D. noxia is found in Australia. Further, from my reading of the manuscript, I understand that one clonal isolate was propagated and then infected with symbionts. I think it is important to reword this sentence so that it is clear that the aphid genetic background is held constant, and that the only differences here are the presence or absence of the different symbionts. My reaction to this sentence was that you are working with the same aphid strain hosting different symbionts.

We have clarified it by adding “the same aphid clone carrying different symbionts” after the different aphid strains.

(3) Measuring symbiont "density" is a tricky thing to do; I explain this above in the public review. I suggest considering some revisions to the manuscript to be sure that you accurately reflect what has been measured and what can reasonably be inferred from using qPCR to measure gene copy number.

We agree that qPCR-based measurements of symbiont gene copy number have limitations. In this experiment, we had a relatively large number of samples, and qPCR provided a practical and efficient approach for assessing variation in endosymbiont abundance and infection status among samples. While we acknowledge the limitations of this approach, the relative differences in gene copy number can still provide an indication of variation in endosymbiont abundance and infection status among treatments. Unfortunately, other approaches remain challenging given resource and expertise limitations.

(4) I think that you may be undervaluing the results of the mixed infection experiments; I find them to be compelling. To me, the data suggest that the symbionts increase aphid fitness.

In our experiments, we assessed aphid fitness under different environmental conditions. In the individual fitness assays conducted on cups with a single wheat plant (Figure 3—figure supplement 1 & Figure 4—figure supplement 1), aphids were maintained under relatively favourable conditions, with limited environmental stress and without substantial crowding. Under these conditions, we observed an increase in fitness associated with symbiont infection. However, we also examined aphid performance under population-level conditions, where populations were established from a small number of aphids and subsequently experienced increasing crowding and density-dependent stress (Figures 3 & 4). Under these conditions, the effects on fitness were different from those observed in the individual assays. We therefore agree that our results suggest that symbionts can increase aphid fitness under some conditions, but that this effect may be context-dependent and can differ under population-level conditions where density-dependent stress occurs. We have also added this information to our Results section to make this clear to readers.

(5) Lines 288-291: This sentence doesn’t make sense to me. What "minor fitness costs" are being referred to? If the infected lines are increasing in representation relative to the uninfected lines, that suggests that there are not fitness costs, but fitness advantages under the experimental conditions.

We have rephrased it to “minor fitness effects” which we refer to the fitness test under favourable conditions.

(6) The section that begins on line 293 - I find this part to not be particularly robust and suggest dropping it from the paper.

We appreciate the reviewer’s concern regarding the robustness of this section. We included this experiment to monitor changes in aphid population over time and to help explain the differences in plant feeding damage observed between aphids carrying different endosymbionts. Importantly, we conducted this experiment using whole wheat plants to evaluate whether the patterns observed in our other experiments were also evident. We believe that these results provide important complementary evidence for interpreting the differences in plant damage among the aphids hosting different endosymbionts and therefore are relevant to the overall conclusions of the study. For this reason, we prefer to retain this section in the manuscript.

(7) Line 298: three plants per time point - I do not think this sample size is reflected in the methods of the paper.

We have mentioned in the method part with “At day 14, three replicate plants were randomly selected from the available plants for both treatments and we counted the total number of nymphs, alate adults, and apterous adults. This was repeated again at Days 21 and 25”.

(8) Line 304: "the frequency of alates decreased as plant damage increased" - this seems to be counterintuitive!

As plant damage increased, the total aphid population also increased, resulting in an increase in the absolute number of alates. However, the frequency (proportion) of alates decreased because the increase in the total aphid population was greater than the increase in the number of alates.

(9) Line 404: "horizontal transmission through plant tissue and/or transfer via aphid contact or honeydew" - what evidence is there that this happens? I have not kept on top of the literature with respect to transmission of secondary symbionts in aphids, but back when I was very familiar with that literature, the data did not support transmission by any of those routes. If there is now evidence supporting transmission by these routes, please cite it here.

Previous studies have provided experimental evidence that horizontal transmission of aphid-associated endosymbionts can occur through plants. For example, plant-mediated transmission has been demonstrated for direct detection of secondary endosymbiont in the plant tissue including *Hamiltonella defensa* (Li et al., 2018), *Rickettsia* (Shi et al., 2024) and *Serratia symbiotica* (Pons, et al., 2019a). Our previous research also demonstrates that *Rickettsiella* endosymbionts were detected in uninfected aphids after feeding by infected aphids regardless of physical contact (Gu et al., 2023). Endosymbionts have also been detected in aphid honeydew (Darby and Douglas, 2003) and some primary transmission route appears to be horizontal, through honeydew (faeces) and host plant phloem (Pons, et al., 2019a & 2019b; Perreau et al., 2021). Appropriate references have been added to the revised manuscript in the Discussion section.

References:

Li et al., Plant-mediated horizontal transmission of *Hamiltonella* defensa in the wheat aphid Sitobion miscanthi. J Agric Food Chem 66, 13367-13377 (2018).

Shi et al., *Rickettsia* transmission from whitefly to plants benefits herbivore insects but is detrimental to fungal and viral pathogens. mBio 15, e02448-23 (2024).

Pons et al., Circulation of the cultivable symbiont *Serratia symbiotica* in aphids is mediated by plants. Front Microbiol 10, 764 (2019a).

Darby and Douglas, Elucidation of the transmission patterns of an insect-borne bacterium. Appl Environ Microbiol 69, 4403-4407 (2003).

Pons et al., New insights into the nature of symbiotic associations in aphids: infection process, biological effects, and transmission mode of cultivable *Serratia symbiotica* bacteria. Appl Environ Microbiol 85, e02445-18 (2019b).

Perreau et al., Vertical transmission at the pathogen-symbiont interface: *Serratia symbiotica* and aphids. mBio 12, e00359-21 (2021).

(10) Line 511: revise to "to measure their relative densities relative to a host gene".

We have revised it.

(11) Throughout the manuscript, please replace "five aged-matched" with an accurate description of the aphids used in the experiment. Please pay particular attention to the figure legends. Simply state e.g. "five 10-day-old apterous females" etc.

This has been replicated in the main manuscript and figure legends.

(9) Line 727 - lowercase t for Tests.

This has been added.

(10) Line 738 - what happens when you don’t exclude the early time points? Do your significant results go away? Also, please define what is meant by "early time points".

When the early time points (Day 14 for *Rickettsiella* and Day 4 for *Regiella*) were included in the analysis, the GLM still showed a significant effect of strain on alate production for *Rickettsiella* (F_1,12_ = 26.434, *P* < 0.001). We excluded these early time points from the analysis presented in the manuscript because, at these stages, aphid population sizes were still similar between treatments. We also conducted independent-sample t-tests at individual time points and observed differences at the later time points, when aphid population sizes began to diverge. We therefore considered the later time points to be more informative for assessing fitness effects and population sizes under increasing crowding conditions. We have now clarified this as “The earliest time points in the experiments for *Rickettsiella* (Day 14) and *Regiella* (Day 4) were excluded” in the manuscript.

(11) In the legends of all figures, please be explicit about sample sizes.

We have added the relevant information about replicate number or sample sizes in the main and supplementary figures.

(12) Line 1010 - replace "each leave" with "each leaf" - there was at least one other place, I think in the supplemental information, that leave was used instead of "leaf".

We replaced this.
